# Prognostic value of microvessel density in cervical cancer

**DOI:** 10.1186/s12935-018-0647-3

**Published:** 2018-10-03

**Authors:** Xiaoli Hu, Hailing Liu, Miaomiao Ye, Xueqiong Zhu

**Affiliations:** Department of Obstetrics and Gynecology, The Second Affiliated Hospital of Wenzhou Medical University, Wenzhou, 325000 Zhejiang China

**Keywords:** Microvessel density, Survival, Cervical neoplasia, Meta-analysis

## Abstract

**Background:**

Several epidemiological researches have indicated that microvessel density (MVD), reflecting angiogenesis, was a negatively prognostic factor of cervical cancer. However, the results were inconsistent. Therefore, we performed a meta-analysis to evaluate the association between microvessel density and the survival probability of patients with cervical cancer.

**Method:**

There was a comprehensive search of the PubMed, EMBASE and Cochrane databases up to August 31, 2017. Based on a fixed-effects or random-effects model, the hazard ratio (HR) and 95% confidence intervals (CIs) were calculated from researches on overall survival (OS) and disease-free survival (DFS).

**Result:**

Totally, we included 13 observational researches, involving 1097 patients with cervical cancer. The results showed that high level of microvessel density was negatively correlated with OS (HR = 1.79, 95% CIs 1.31–2.44, *I*^2^ = 60.7%, *P* = 0.003) and DFS (HR = 1.47, 95% CIs 1.13–1.80, *I*^2^ = 0%, *P* = 0.423) of cervical cancer patients. In subgroup analysis, high counts of MVD were significantly associated with a poor survival (including OS and DFS) of the patients detected by anti-factor VIII antibodies or in European origin.

**Conclusion:**

The present meta-analysis indicated that survival with high level of MVD was significant poorer than with low MVD in cervical cancer patient. Standardization of MVD assessment is needed.

## Background

Cervical cancer is the third most frequent gynecological neoplasms worldwide and one of the leading causes of cancer-related death among women in developing countries [1]. This disease is responsible for approximately 265,000 deaths annually in the world, 87% occurring in low-income countries [2]. Although independent prognostic factors such as lymph node status, tumor size, pathologic grading of tumor and International Federation of Gynecology and Obstetrics (FIGO) stage contribute to a better comprehension of the disease progression [3, 4]. However, such factors couldn’t predict individual clinical outcome absolutely in cervical cancer. Thus, there need more prognostic markers to further improve predictive accuracy.

Angiogenesis have been reported to play a crucial role in the growth, metastasis and progression of various types of cancers like breast cancer and renal cell carcinoma [5, 6]. When a tumor exceeds the size of 1 mm, its further growth needs angiogenesis, which can form new blood vessels and further lead to tumor metastasis. There are a variety of biomarkers to quantify intratumoral angiogenesis, including vascular endothelial growth factor (VEGF), basic fibroblast growth factor (bFGF) and microvessel density (MVD).

At present, MVD assessment is the most common method to evaluate intratumor angiogenesis in cancer. In early 1990s, MVD was firstly introduced as an indicator by Weidner et al. [7] to assess the microvessels density in patients with invasive breast cancer. Additionally, the most commonly used antibodies for microvessel staining are CD31, CD34, CD105 (Endoglin) and anti-factor VIII (Von Willebrand Factor). Over the past two decades, some studies had reported MVD as a prognosis factor in various tumors such as gastric carcinoma [8].

However, the value of microvessel density as a prognostic indicator of cervical cancer was controversial. Several researches demonstrated that the expression of MVD significantly associated with poor overall survival (OS) or progression free survival (PFS) for cervical cancer [9, 10]. But some studies were unable to indicate a significant relationship between MVD and poor survival in cervical cancer patients [11, 12].

Therefore, we conducted a meta-analysis in order to evaluate the prognostic value of microvessel density in patients with cervical carcinoma. Meanwhile, this study may help to provide a valuable prognostic indicator and guide the management of the cervical cancer patients in the future.

## Methods

### Search strategy

Two investigators conducted a comprehensive search in electronic databases of PubMed, EMBASE and Cochrane library for relevant researches up to 31 August 2017. The following Medical Subject Heading terms and keywords were used: (“cervical cancer” or “cervical tumor” or “cervical tumour” or “cervical malignance” or “cervical carcinoma” or “uterine cervical neoplasms” or “cervical neoplasm”) and (“microvessel density” or “MVD” or “angiogenesis” or “neovascularization”) and (“prognosis” or “outcome” or “survival” or “prognostic”) with no restrictions. Meanwhile, we also performed a manual search of references cited in the retrieved studies and published reviews.

### Selection criteria

To be eligible, researches must be consistent with the following inclusion criteria: (1) All included patients with cervical carcinoma diagnosed by the pathological results; (2) reported the association between MVD and survival outcomes, such as overall survival (OS) and disease free survival (DFS); (3) papers were restricted to human studies published as full-length articles in English. Exclusion criteria were (1) reviews, letters, case reports, or editorial comments; (2) duplicate publications; (3) full text unavailable; (4) insufficient data for calculating the hazard ratios (HRs) and 95% confidence intervals (CIs).

The candidate studies were identified by two independent reviewers according to the titles and abstracts to exclude irrelevant studies. Then full texts of the remaining researches were scanned carefully to decide whether to include the studies, and any different opinion was resolved through discussion. Multivariate data were the priority choice when both multivariate and univariate data were offered. However, we also accepted univariate data when no multivariate results were provided.

### Data extraction and quality assessment

The following information were extracted carefully from all including studies by two authors by means of a standardized data table which included following items: the first author; the year of publication; the location of study; the number of included patients; the age range of participants; FIGO stage; the antibody to assess MVD; the duration of follow up; the cutoff value of MVD (usually with median MVD as cutoff); the types of survival analyses; HRs and 95% CI for overall survival/disease-free survival; the result of the study. The result for every single study was marked “positive” when higher MVD predicted poorer survival and “negative” when higher MVD did not indicate lower survival rate or even once supported a better survival.

Quality assessment of the including studies was evaluated by two investigators independently using the 9-star Newcastle–Ottawa Scale (NOS) [13]. According to the scoring system, we defined the research quality as high with scores which were equal to or greater than 7.

### Statistical analysis

The prognostic efficiency of MVD on cervical carcinoma was calculated by using the HRs and 95% CIs. When the effect values couldn’t be provided directly by the study, we calculated HR value and 95% corresponding CIs in the Kaplan–Meier curve at particular time points using the methods introduced by Parmar et al. [14]. An observed HR > 1 indicated a bad prognosis in cervical cancer patient with the high MVD. Statistical heterogeneity from all the publications was tested by Cochran’s Q test and Higgins I-squared statistics [15]. Meanwhile, a fixed-effects model was adopted to assess the pooled value when *I*^*2*^ < 50% and *P* > 0.10 which indicated that no obvious heterogeneity was found. Otherwise, a random-effects model was applied. All statistical analyses were performed with STATA 11.0 (STATA Corp, College Station, Texas).

In addition, Subgroup analyses were conducted to calculate the potential source of heterogeneity according to geographical regions and antibodies for detecting MVD. A sensitivity analysis evaluating the consistency of the combined outcomes was adopted. The possible publication bias was assessed by Begg’s tests [16]. All P-value were two-tailed and statistical significance was obvious as *P* < 0.05.

## Results

### Literature search

**Fig. 1:**
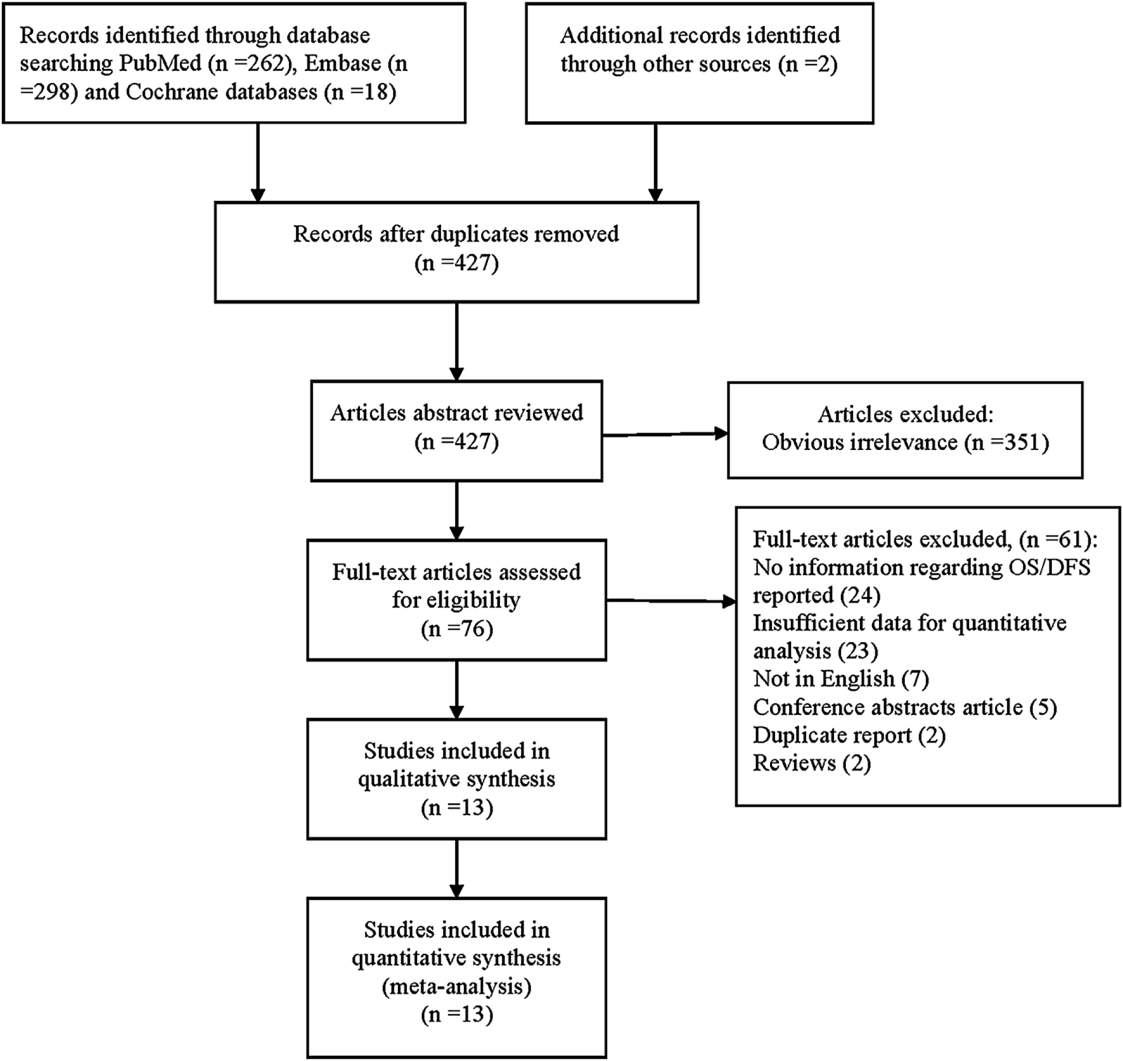
Flow diagram for study selection

At the beginning, there were a total of 580 articles identified from three databases (262 from PubMed, 298 from EMBASE, 18 from Cochrane databases) according to the inclusion criteria. After screening the title, abstract and key words, 427 articles were deleted when met duplication. Followed by excluded researches which were obvious irrelevant, or didn’t meet the inclusion criteria, we adopted a number of 13 independent and observational studies involving 1097 patients [9–11, 17–26]. The flow chart presented in Fig. 1 showed the study selection process in detail.

### Study characteristics

Table 1 presented the main characteristics of the 13 studies included in the meta-analysis. These researches were published between 1995 and 2014, and the sample sizes ranged from 30 to 173. As for the region, three studies were conducted from America [9, 21, 24], seven in Europe [10, 17, 18, 22, 23, 25, 26] and three in Asian country [11, 19, 20]. In addition, there were four different antibodies enrolled in the included studies to assess the microvessel density. Anti-factor VIII antibody was used in six studies [10, 18–20, 22, 26], CD 31 was conducted in four studies [17, 21, 23, 24], CD 34 was in two articles [9, 11], CD 105 was applied in two studies [21, 23], respectively. And all of the protein-levels of antibody were detected by immunohistochemistry (IHC). Among them, MVD count in ten studies was performed by hotspot method which was introduced by Weidner et al. [7], while the hotspot method was not mentioned in other three articles. Thus, 14 researches (from 13 articles) involving 1097 patients were available for this meta-analysis. We included 11 studies which gave a description of the association between OS and MVD, and six researches involved other outcomes such as DFS. Otherwise, we defined the study as ‘− 1’ and ‘− 2’ if more than one outcome or antibody were applied in the same study [27, 28]. Meanwhile, the quality of included researches were assessed by using the Newcastle–Ottawa Scale and found to range from 7 stars to 9 stars, showing that the studies were in high quality.

**Table 1:** Main characteristics of included studies

Study (year, population)	Age	FIGO stage	Sample size	Antibody	Follow-up (month)	Method for MVD	Cutoff of MVD	HR (95% CIs)	Survival analysis	Results	NOS
Cantu et al. (2003, Mexico) [9]	48 (28–70)	II–III	118	CD34	60 (median)	Hot spot	20	DFS: 1.63 (1.71–2.65)	DFS	Positive	7
Cooper et al. (1998, England) [10]	50 (29–81)	I–III	111	Anti-factor VIII	55 (28–117) (median)	Hot spot	10	OS: 1.68 (1.03–2.74); DFS: 2.2 (1.09–4.44)	OS, DFS	Positive	8
Dellas et al. (1997, Switzerland) [17]	NR	IB	58	CD31	67 (median)	Hot spot	NR	OS: 1.5 (1.04–2.16)	OS	Positive	7
Hockel et al. (2001, Germany) [18]	NR	IB1–VIB	52	Anti-factor VIII	26 (4–96) (median)	Hot spot	40	OS: 3.17 (1.32–7.63); DFS: 2.08 (1.22–3.54)	OS, DFS	Positive	8
Kaku et al. (1998, Japan) [19]	46 (25–67)	I–II	56	Anti-factor VIII	99 (61–175) (median)	Hot spot	75	OS: 3.31 (1.21–9.07)	OS	Positive	8
Lee et al. (2006, Korea) [11]	48 (31–69)	IB–IIB	85	CD34	60	IHC	30.5	OS: 1.11 (0.63–1.98); DFS: 1.04 (0.59–1.85)	OS, DFS	No significance	9
Moriyama et al. (2009, Japan) [20]	55.9 (33–72)	IB–IIB	57	Anti-factor VIII	10.5–174.3	Hot spot	0.8%	OS: 2.92 (1.11–7.69)	OS	Positive	8
Randall et al. (2009, America) [21]	39.1	IA2, IB, IIA	173	CD31, CD105	105.9 (2.7–184.8) (median)	Hot spot	110 in CD31, 28 in CD105	CD31-OS: 0.36 (0.17–0.79); CD105-OS: 1.76 (0.89–3.48)	OS	Positive in CD31	8
Schlenger et al. (1995, Germany) [22]	53 (26–80)	IB–IVA	39	Anti-factor VIII	18 (4–41) (median)	IHC	83	DFS: 1.46 (1.05–2.03)	DFS	Positive	8
Tjalma et al. (1998, Belgium) [25]	54 (24–91)	IA–IVB	114	CD31	39 (1–203)	Hot spot	261	OS: 2.17 (1.15–4.1)	OS	Positive	7
Van et al. (2006, America) [24]	42 ± 11 (26–79)	IB–IVA	38	CD31	17 ± 17 (1–71) (mean)	IHC	NR	OS: 2.54 (1.11–5.82)	OS	Positive	7
Zijlmans et al. (2009, Netherlands) [23]	45 (29–72)	IB–VIB	30	CD105	NR	Hot Spot	NR	OS: 3.81 (0.46–31.42); DFS: 3.23 (1.38–7.57)	OS, DFS	Positive	8
Obermair et al. (1998, Austria) [26]	43 (23–70)	IB	166	Anti-factor VIII	85 (5–170) (median)	Hot Spot	20	OS: 2.56 (1.59–4.11)	OS	Positive	7

*HR* hazard ratio, *CIs* confidence intervals, *NA* not available, *OS* overall survival, *DFS\PFS\MFS\RFS* disease-free survival/progress-free survival/metastasis-free survival/recurrence-free survival

### Association of MVD and OS of cervical cancer

**Fig. 2:**
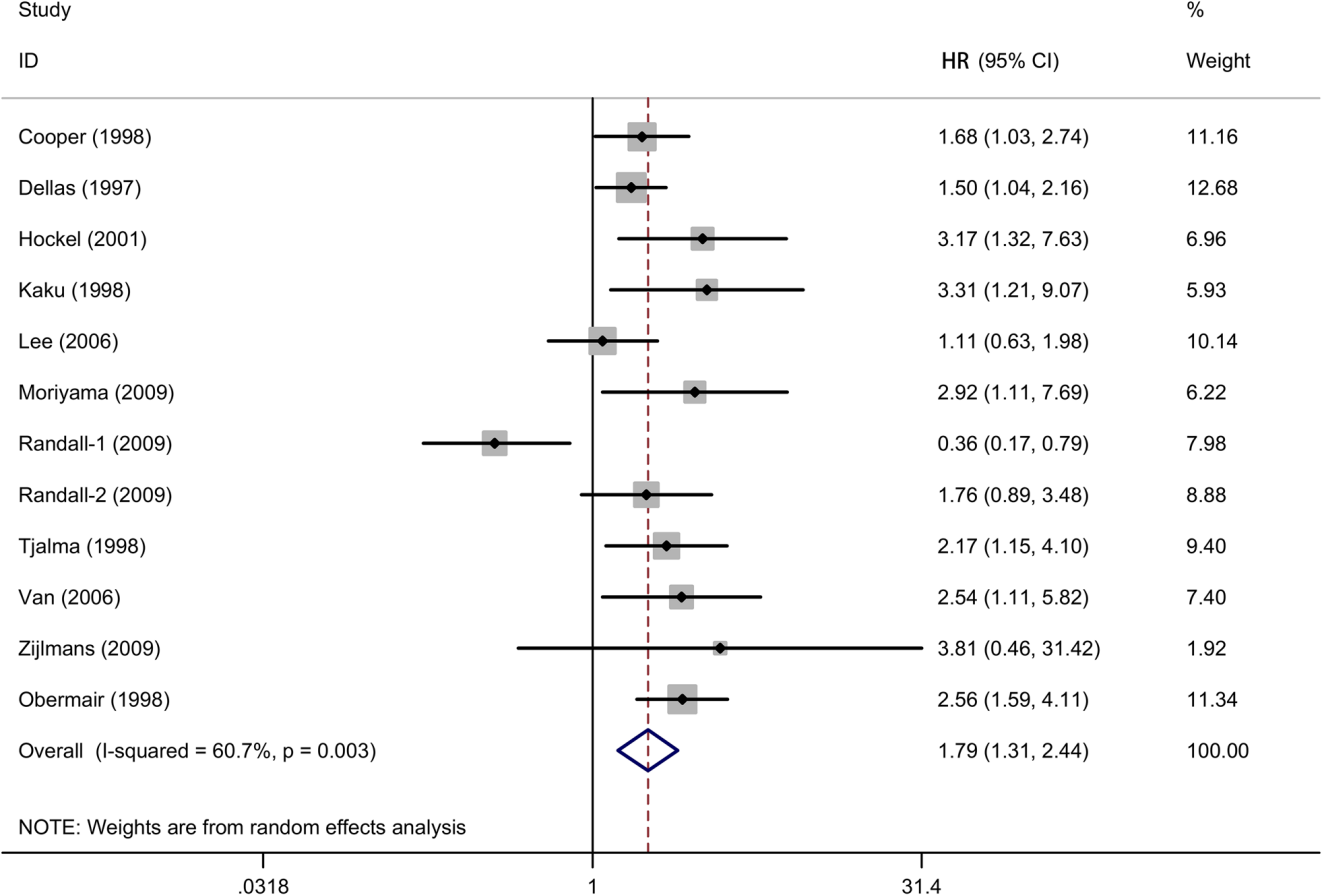
The forest plot assesses the association between MVD and cervical cancer with OS

**Fig. 3:**
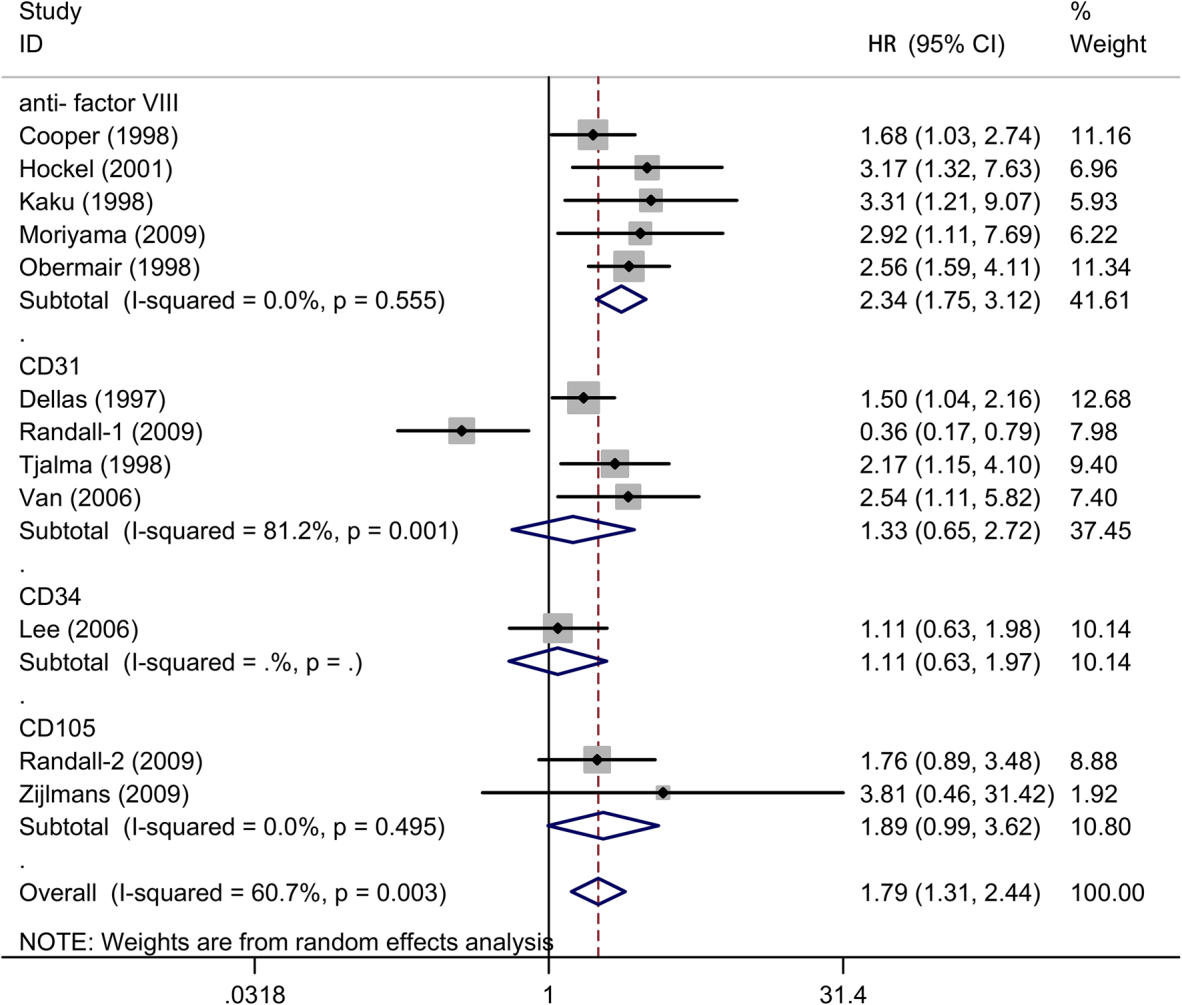
Subgroup analysis of association between count of MVD and prognosis of cervical cancer with OS detected by different antibodies

**Fig. 4:**
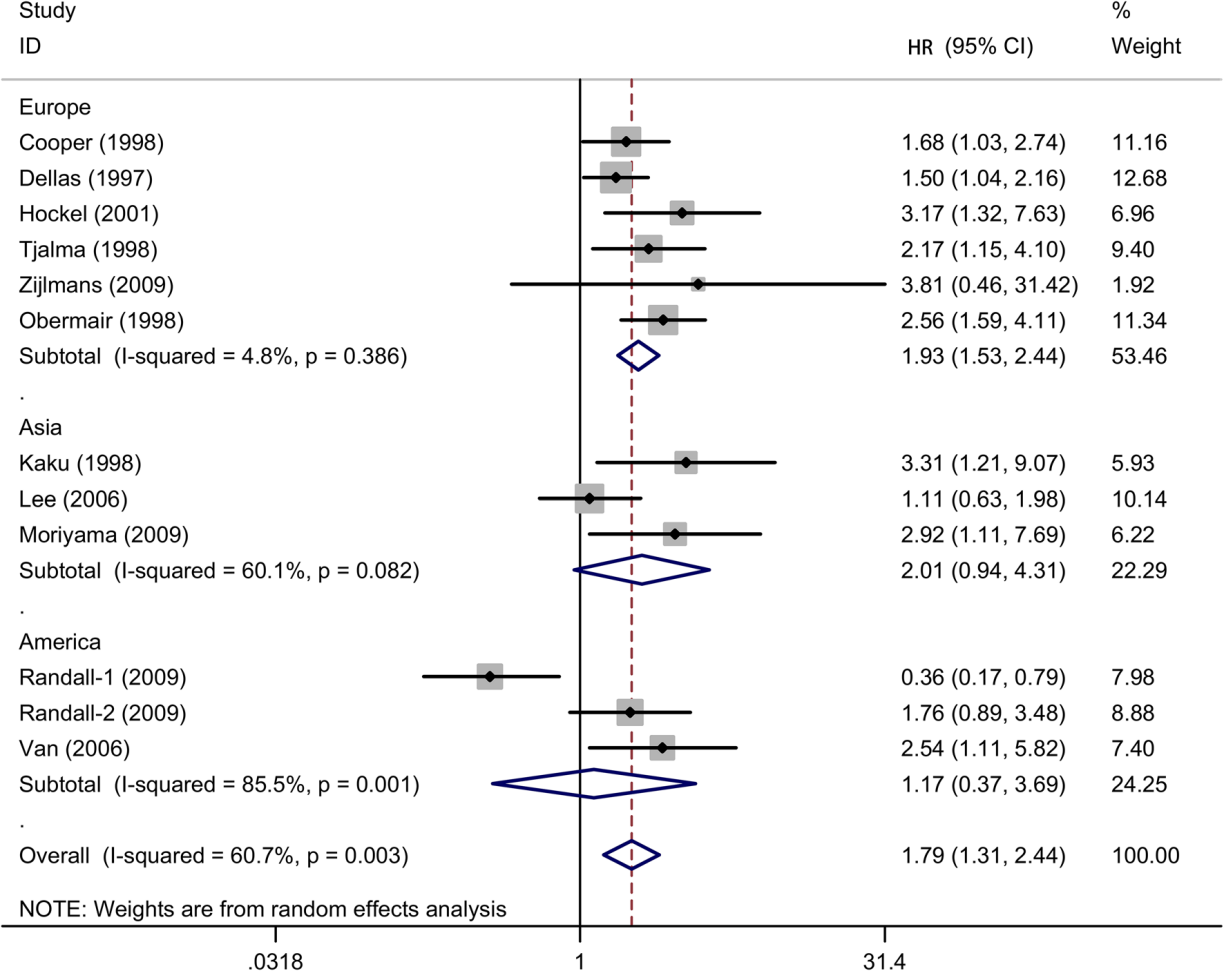
Subgroup analysis of association between count of MVD and prognosis of cervical cancer with OS in different populations

The pooled HR for the 11 studies assessing the association between MVD and cervical cancer with OS was 1.7 (95% CIs 1.31–2.44, random-effects, Fig. 2), suggesting that high MVD level was associated with a poor prognosis of overall survival in cervical cancer patients. Since the heterogeneity among studies was significant *(I*^2^ = 60.7%, *P* = 0.003), random-effects model was applied for statistical analysis. Meanwhile, subgroup meta-analysis according types of antibodies and population was conducted to evaluate the possible source of heterogeneity among these studies (Figs. 3 and 4).

In the subgroup analysis by different antibodies, the prognostic value of MVD for OS was significant in the “anti-factor VIII” subgroup (HR = 2.34, 95% CIs 1.75–3.12, *I*^2^ = 0%, n = 5), while there was not statistically significant association in “CD31” subgroup (HR = 1.33, 95% CIs 0.65–2.72, *I*^2^ = 81.2%, n = 4), “CD34” subgroup (HR = 1.11, 95% CIs 0.63–1.97, n = 1) and “CD105” subgroup (HR = 1.89, 95% CIs 0.99–3.62, *I*^2^ = 0%, n = 2).

There was another subgroup about source regions of included studies, a pooled HR was 1.93 (95% CIs 1.53–2.44, *I*^2^ = 4.8%, n = 6) in Europe population, indicating a significantly poorer survival in cervical cancer patients with higher MVD in European countries. However, MVD level was not significantly associated with OS in Asia (HR = 2.01, 95% CIs 0.94–4.31, *I*^2^ = 60.1%, n = 3) and American locations (HR = 1.17, 95% CIs 0.37–3.69, *I*^2^ = 85.5%, n = 3).

### Association of MVD and DFS of cervical cancer

**Fig. 5:**
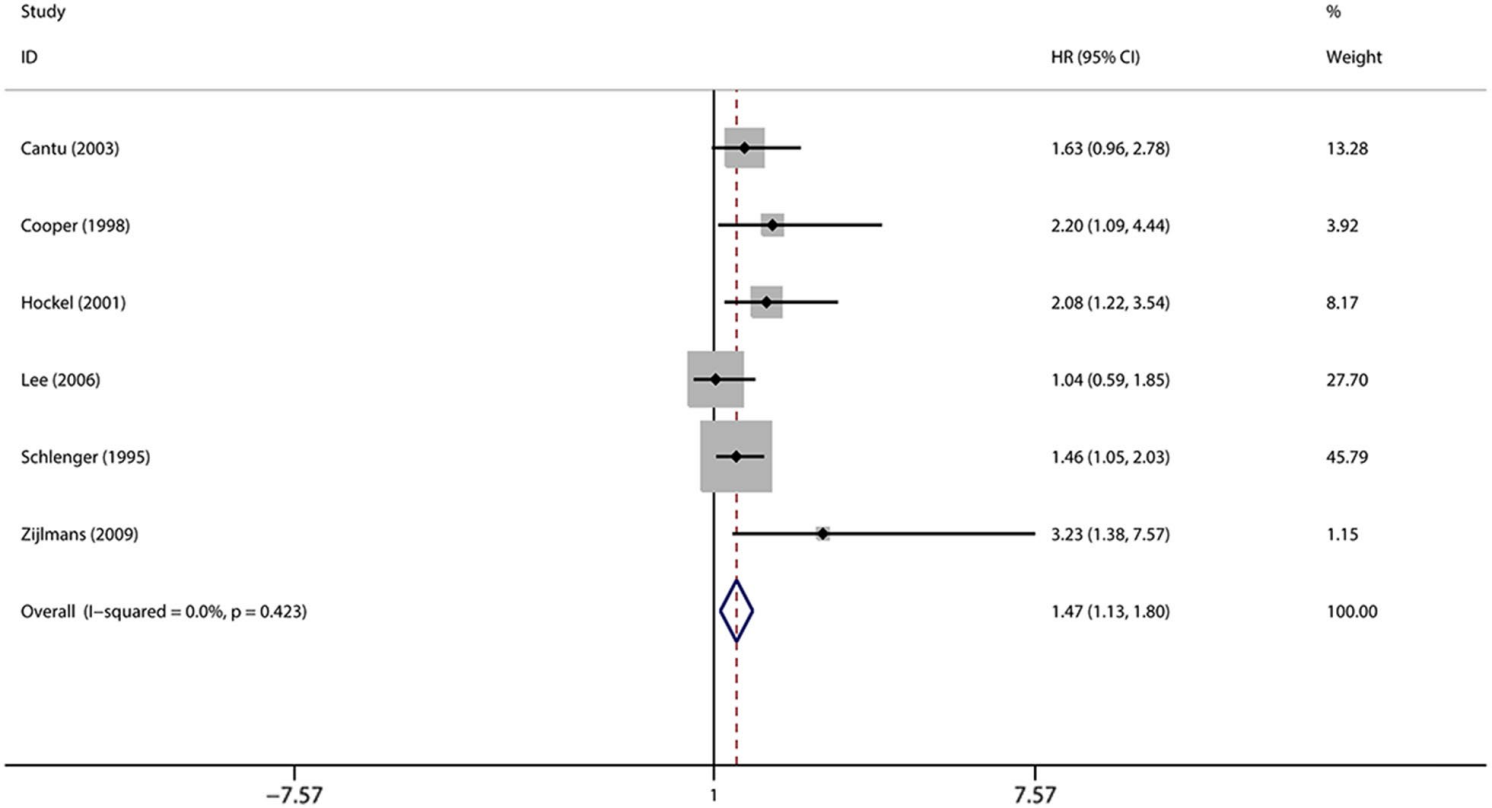
The forest plot illustrates the association between MVD and DFS of cervical cancer

We analyzed the relationship between the level of MVD and DFS among cervical cancer patients. There was no heterogeneity of data (*I*^2^ = 0%), in which a fixed-effect model was selected to assess the pooled outcome (Fig. 5). As a result, MVD level was associated with a worse DFS of cervical cancer patients (HR = 1.47, 95% CIs 1.13–1.80, n = 6).

**Fig. 6:**
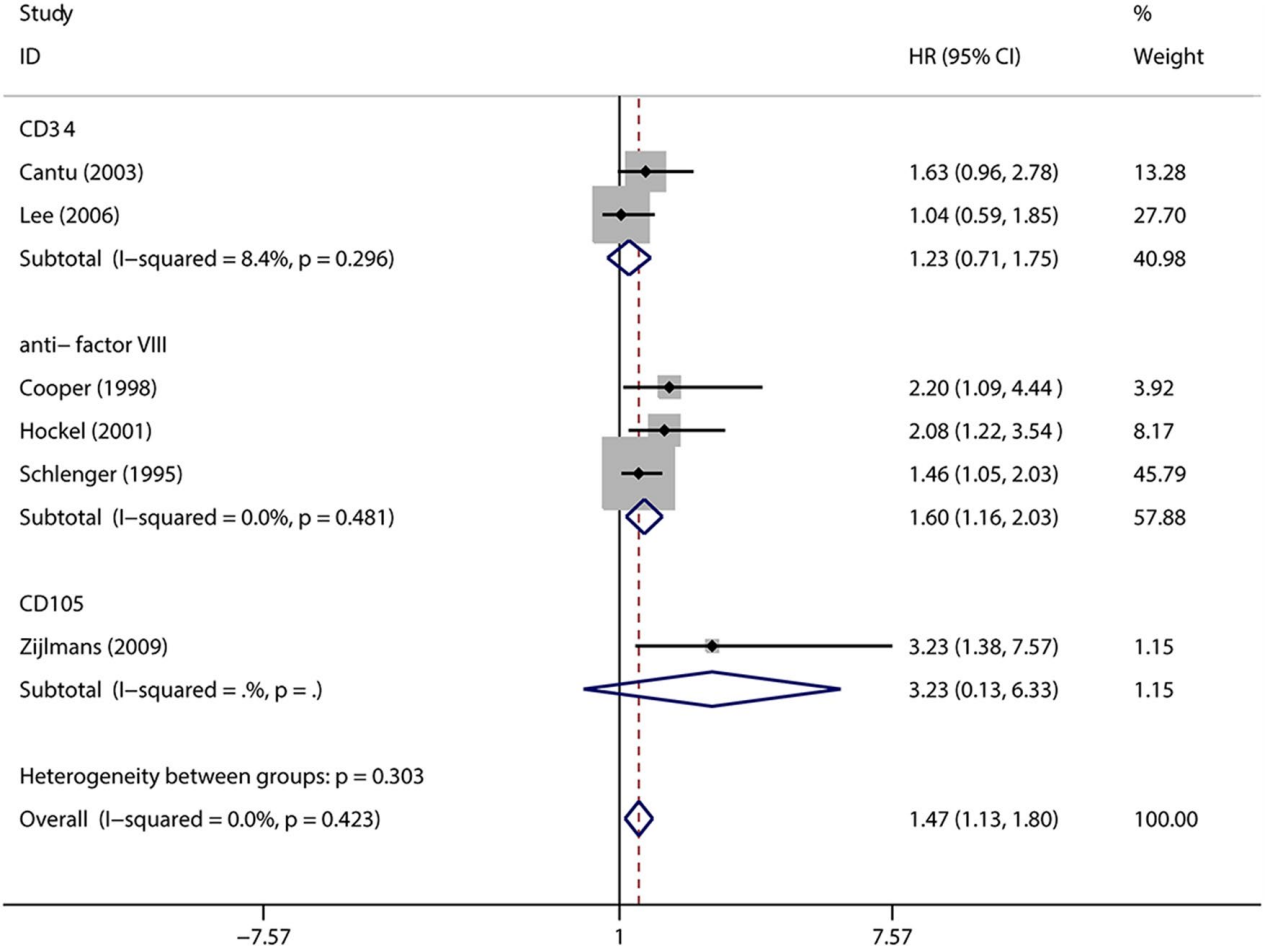
Subgroup analysis of association between count of MVD and prognosis of cervical cancer with DFS detected by different antibodies

**Fig. 7:**
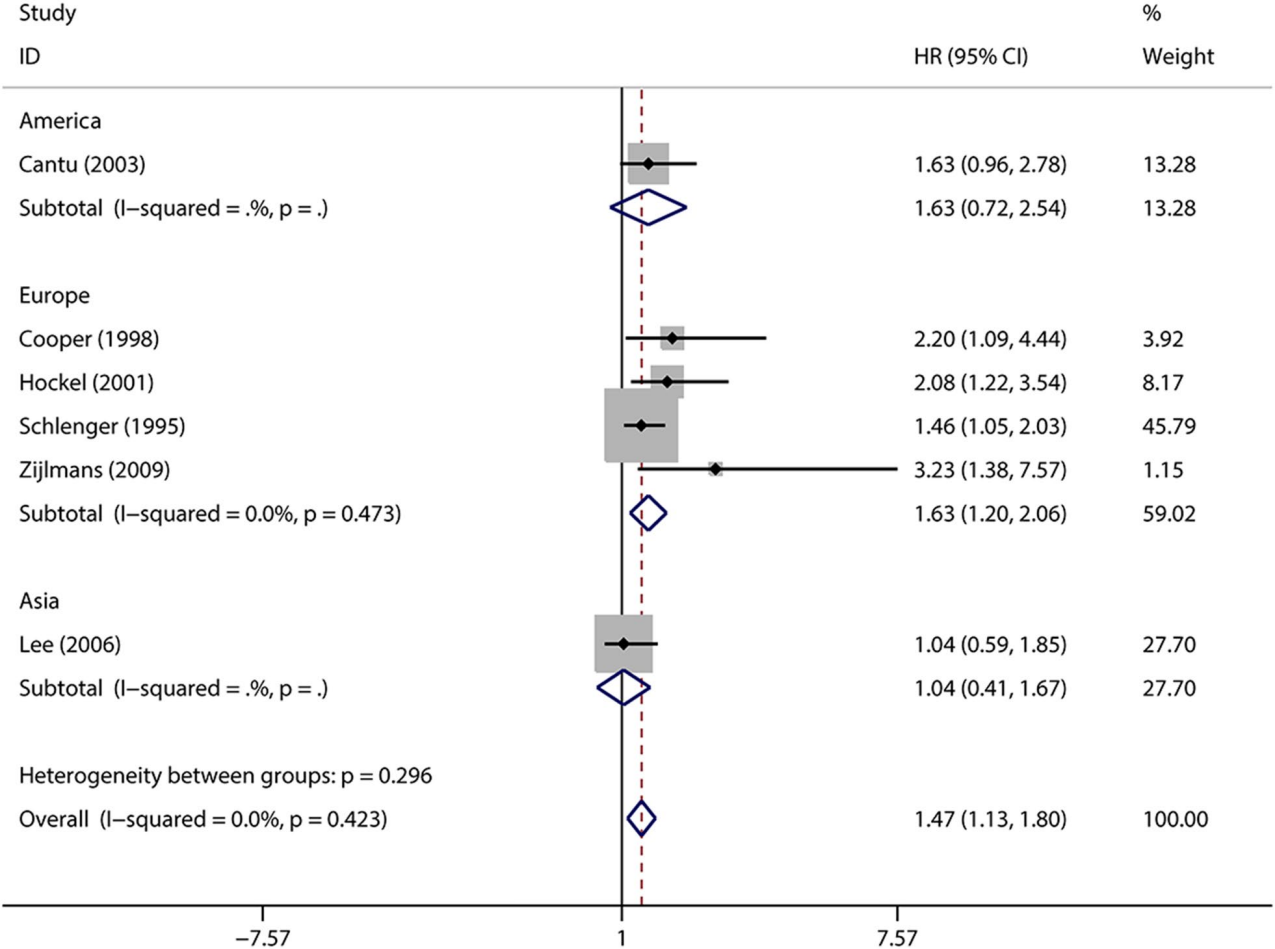
Subgroup analysis of association between count of MVD and prognosis of cervical cancer with DFS in different populations

Furthermore, subgroup analyses based on antibody and region were used to explore the influencing factors which may impacted the overall outcomes (Figs. 6 and 7). Divided by different immunohistochemical biomarkers among the subgroups, “anti-factor VIII” antibody (HR = 1.60, 95% CIs 1.16–2.03, *I*^2^ = 0, n = 3) showed the significantly negative association between MVD and DFS among cervical cancer patients, but not in “CD34” subgroup (HR = 1.23, 95% CIs 0.71–1.75, *I*^2^ = 8.4%, n = 2).

In addition, the included studies were stratified into the three regional distribution of patients (Europe, Asia and America). Negative effected of MVD on DFS in European countries were found in patients with cervical cancer (HR = 1.63, 95% CIs 1.20–2.06, *I*^2^ = 0%, n = 4), but not in Asia location (HR = 1.47, 95% CIs 1.13–1.80, *I*^2^ = 0%, n = 2).

### Sensitivity analysis

**Fig. 8:**
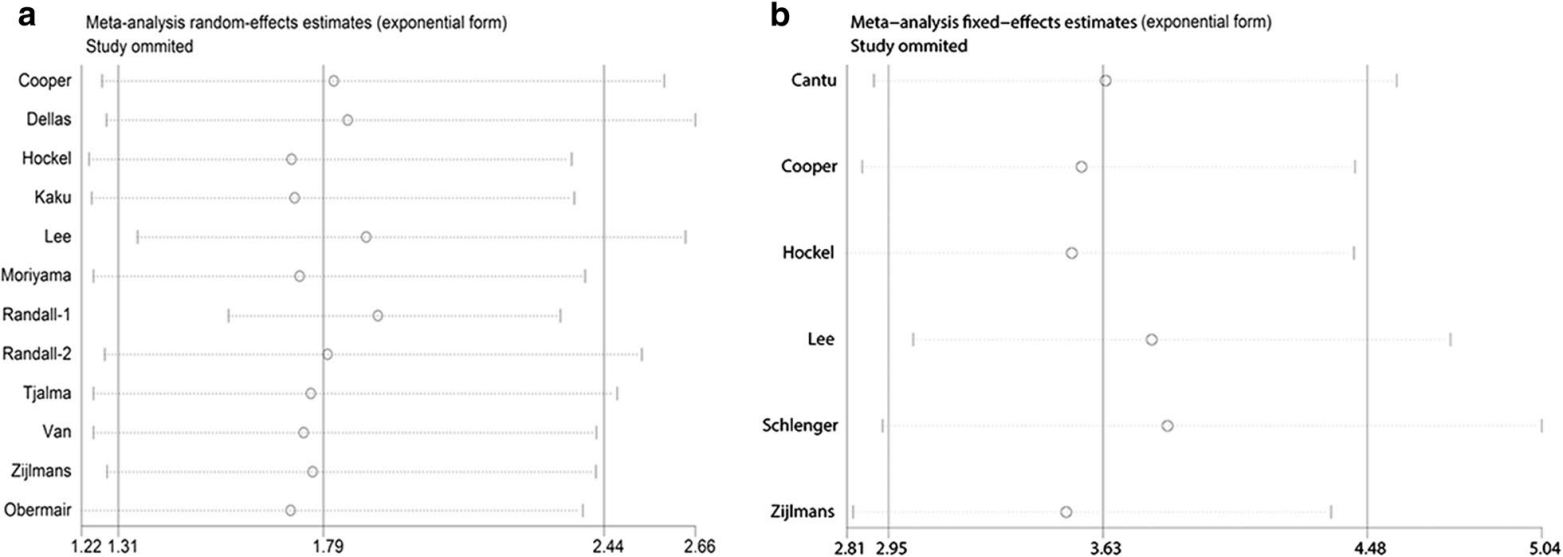
Sensitivity analysis to assess the stability of the pooled outcomes between count of MVD and prognosis of cervical cancer. **a** OS; **b** DFS

In sensitivity analysis, the leave-one-out method was applied to assess the stability of the pooled outcomes. Eligible studies were sequentially removed one by one to evaluate the influence of each included study on the overall HR. After leaving out any single study, statistical significance of the OS or DFS did not change, suggesting no individual study had excessive influence of the association of MVD and cervical cancer (Fig. 8a, b).

### Publication bias

**Fig. 9:**
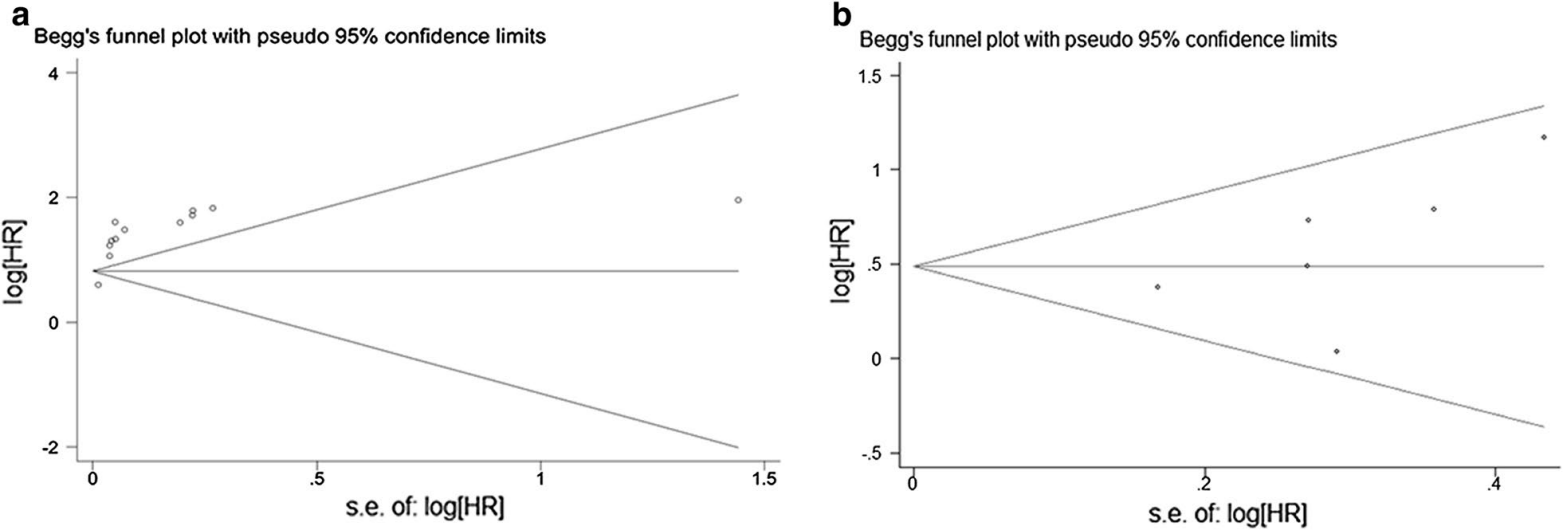
The presence of publication bias for the overall relationship between MVD level and the prognosis of cervical cancer. **a** OS; **b** DFS

The presence of publication bias for the overall relationship between MVD level and the prognosis of cervical was calculated by using the Begg’s rank correlation test (*P* = 0.15 for OS; *P* = 0.238 for DFS). Funnel plots were graphically symmetric, indicating that there was no significant publication bias among the included articles (Fig. 9a, b).

## Discussion

A number of studies have showed that MVD played the potential role as prognostic biomarker for many cancers. In our present meta-analysis, we confirmed that high counts of MVD, a marker of angiogenesis, was significantly associated with worse prognosis in patients with cervical cancer. Moreover, results were also conducted in subgroup analyses for patients detected by different antibodies or in various countries. Although some modest bias cannot be excluded, this was the first meta-analysis of published articles to assess the relationship between MVD count and prognosis in cervical cancer.

Between-study heterogeneity was significant in our meta-analysis for OS (*I*^2^ = 60.7%). However, there was no heterogeneity for DFS (*I*^2^ = 0%). We tried to reduce the variability by screening the researches using the same standard, which was to divide studies into different subgroups, such as the regional distributions and staining markers. However, the heterogeneity could not be eliminated in general. But the heterogeneity had decreased in some subgroups such as the Europe group (*I*^2^ = 4.8% and 11.8%) and the group using anti-factor VIII as biomarkers on OS (*I*^2^ = 0%) for the MVD group. These results showed that all the different factors played important roles in the generation of heterogeneity which couldn’t be eliminated at the same time.

Obviously, our study showed that the selection of antibody as a biomarker for MVD assessment played an important role for conclusion. There were eight studies in our meta-analysis using factor VIII as an endothelial biomarker, eight studies using other antibodies such as CD31, CD34 and CD105. We have established that the counts of MVD assessed by factor VIII were significantly related with the poor outcomes of cervical cancer, including OS and DFS. However, there was no statistical significance in the association between the levels of MVD assessed by anti-CD31, CD34 or CD105 with the prognosis of cervical cancer patients for OS or DFS. Weider et al. [7] chose an antibody against factor VIII-related antigen to mark mainly the endothelia of mature vessels. This biomarker was still the most commonly used in the related studies about microvessel density, however factor VIII did not express in all endothelial cells. And factor VIII also expressed in lymphatic endothelium and platelets, which would result in cross-reactivity with non-endothelium [29]. At present, for these antibodies expressed in lymphatic endothelium and platelets leading to appropriate marker for MVD in tumor has not been established. Recently, other antibodies were used to assess the counts of MVD in cervical cancer [30, 31], such as CD31 (also known as platelet endothelial cell adhesion molecule), CD34 and CD105 (also known as Endoglin). It indicated that CD34 had an improved sensitivity and specificity than factor VIII for endothelial cells activated by regional tumor angiogenesis [32]. Uzzan et al. [27] found that the microvessel counting evaluated by anti-CD31 or anti-CD34 were approximately 30% higher than factor VIII. Therefore, we considered that no statistical significance of relationship between counts of MVD assessed by other biomarkers including CD31, CD34 and CD105, and prognosis of cervical cancer patients was limited by a few number of related studies. Thus, more high-quality studies concerning the MVD detecting and MVD count should be acquired in future. Meanwhile, we found that there were more researches about the relation between MVD and prognosis of cervical cancer patients in Europe group than in Asia and America. When stratified by geographical area, our subgroup analysis indicated that patients with cervical cancer who had a higher level of MVD would had the poorer survival in Europe countries, while the results could not be verified in Asia and America. It was probably because of the insufficient quantity in above-mentioned two regions.

In addition, several limitations of this meta-analysis should also be discussed. First of all, the choice of cutoff values for high MVD varied among the studies, some included articles mainly used median level, while others applied mean or even an inaccurate bound. These differences were responsible for the difficulty in determining a standard cut off value in clinical practice. Therefore, future researches should aim to standardize MVD assessment method. Secondly, for HRs couldn’t be provided directly or calculated from the data in some studies, we need to extract the data from survival curve graphs. Furthermore, it is inevitable that the patient’s baseline status in included studies were different, such as age, menopausal status, tumor type, tumor size, lymph node status, the immunohistochemical marker for MVD detecting and duration of follow-up. Finally, all the included studies in our meta-analysis were retrospective observational researches, more prone to bias than randomized controlled trails.

## Conclusions

To conclude, despite above-mentioned limitations, our meta-analysis strongly showed a poor survival of high counts of MVD in patients with cervical cancer, including OS and DFS, respectively. Moreover, these MVD-related biomarkers could be further used in the prognosis prediction of cervical cancer in clinical practice. However, future basic researches and randomized controlled studies with large samples are needed to conduct the prognostic value of MVD for patients with cervical cancer.

## Abbreviations

MVD: microvessel density
HR: hazard ratio
CIs: confidence intervals
OS: overall survival
DFS: disease-free survival
FIGO: International Federation of Gynecology and Obstetrics
VEGF: vascular endothelial growth factor
bFGF: basic fibroblast growth factor
NOS: the 9-star Newcastle–Ottawa Scale
IHC: immunohistochemistry
NA: not available
